# A metallo-β-lactamase enzyme for internal detoxification of the antibiotic thienamycin

**DOI:** 10.1038/s41598-021-89600-x

**Published:** 2021-05-12

**Authors:** Seydina M. Diene, Lucile Pinault, Sophie Alexandra Baron, Saïd Azza, Nicholas Armstrong, Linda Hadjadj, Eric Chabrière, Jean-Marc Rolain, Pierre Pontarotti, Didier Raoult

**Affiliations:** 1 IRD, APHM, MEPHI, IHU-Méditerranée Infection, Aix Marseille Univ, 19-21 Boulevard Jean Moulin, 13005 Marseille, France; 2 Publique-Hôpitaux de Marseille (AP-HM), IHU-Méditerranée Infection, Marseille, France; 3 IHU-Méditerranée Infection, Marseille, France; 4 CNRS, Marseille, France

**Keywords:** Antibiotics, Bacterial toxins, Antimicrobial resistance

## Abstract

Thienamycin, the first representative of carbapenem antibiotics was discovered in the mid-1970s from soil microorganism, *Streptomyces cattleya*, during the race to discover inhibitors of bacterial peptidoglycan synthesis. Chemically modified into imipenem (N-formimidoyl thienamycin), now one of the most clinically important antibiotics, thienamycin is encoded by a thienamycin gene cluster composed of 22 genes (*thnA* to *thnV*) from *S. cattleya* NRRL 8057 genome. Interestingly, the role of all *thn*-genes has been experimentally demonstrated in the thienamycin biosynthesis, except *thnS*, despite its annotation as putative β-lactamase. Here, we expressed *thnS* gene and investigated its activities against various substrates. Our analyses revealed that ThnS belonged to the superfamily of metallo-β-lactamase fold proteins. Compared to known β-lactamases such as OXA-48 and NDM-1, ThnS exhibited a lower affinity and less efficiency toward penicillin G and cefotaxime, while imipenem is more actively hydrolysed. Moreover, like most MBL fold enzymes, additional enzymatic activities of ThnS were detected such as hydrolysis of ascorbic acid, single strand DNA, and ribosomal RNA. ThnS appears as a MBL enzyme with multiple activities including a specialised β-lactamase activity toward imipenem. Thus, like toxin/antitoxin systems, the role of *thnS* gene within the thienamycin gene cluster appears as an antidote against the produced thienamycin.

## Introduction

Bioactive compounds such as secondary metabolites are biosynthesised by non-ribosomal peptide synthetases (NRPS) and polyketide synthases (PKS), enzymes that have been identified in the different domains of life with an extraordinary diversity and more than 3,300 NRPS/PKS have been reported from 991 different organisms^1^. These bioactive compounds exhibiting a wide range of biological activities can include siderophores, pigments, cytostatics, immunosuppressants, toxins, and antibiotics^2,3^. Toxins or natural antibiotics are expressed by some microorganisms (e.g. fungus and/or bacteria) to master their ecosystems^4^. According to the mode of action, antibiotics can be classified into several groups including synthesis inhibitors of cell walls, proteins, DNA, RNA, and others^5^. Most antibiotic classes including aminoglycosides, macrolides, tetracyclines, chloramphenicol, and β-lactams have been synthesised by biosynthesis gene clusters identified in *Actinobacteria* such as *Nocardia*, *Saccharopolyspora*, *Kitasatospora*, or *Streptomyces*^5^. Furthermore, some β-lactams, especially carbapenem antibiotics, can be synthesised by more common bacteria such as Enterobacterial phytopathogens, including *Serratia sp*. or *Erwinia carotovora* subsp. *atroseptica* (now renamed *Pectobacterium carotovora* subsp. *atroseptica*), or *Photorhabdus luminescens*^6,7^. Like toxin-antitoxin systems, these microorganisms develop self-resistance mechanisms to protect themselves against attacks from their own biosynthetic bioactive compounds^5,8^. These self-resistance mechanisms such as antibiotic efflux pumps, antibiotic-modifying enzymes, or antibiotic-hydrolysing enzymes are often encoded by genes located within the biosynthesis gene clusters^5^. Among these antibiotics, thienamycin, the first to be discovered and characterised as a carbapenem and one of the most clinically important antibiotic classes, is encoded by a thienamycin gene cluster (TGC) composed of 22 genes (*thnA* to *thnV*) from *Streptomyces cattleya* NRRL 8057 genome^9,10^. In addition to thienamycin, another antibiotic. cephamycin (cephalosporin sub-class) encoded by a cephamycin gene cluster (CGC) (composed of 16 genes), has been also identified within the same *S. cattleya* NRRL 8057 genome^10^. However, while the role of almost all genes of the TGC have been experimentally well characterised in thienamycin biosynthesis^11,12^, the role and function of the *thnS* gene, despite its annotation as putative β-lactamase, remain unreported and it was suggested this should be clarified^12^. Here, we investigate the phylogenetic relationship of the ThnS enzyme with bacterial β-lactamase enzymes and explore its enzymatic activity against different substrates including antibiotic drugs, ascorbic acid, DNA, and RNA substrates.

## Results

### Origins of ThnS enzyme

**Figure 1:**
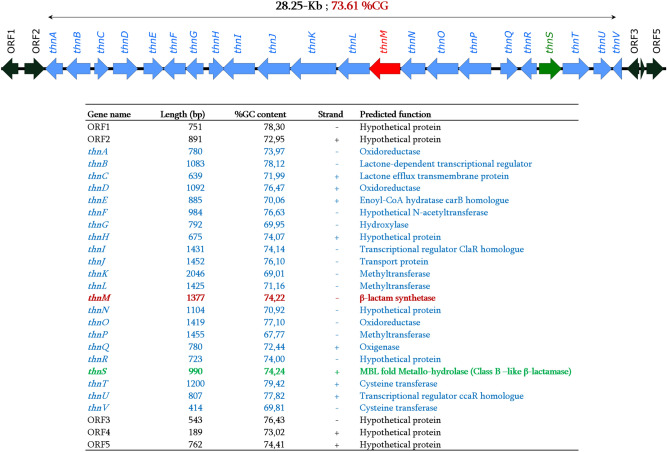
Genetic environment of the thienamycin gene cluster from the megaplasmid of *Streptomyces cattleya* NRRL8057.

The *thnS* gene is part of the thienamycin gene cluster (TGC) located on the linear megaplasmid (pSCAT, 1′809′491-bp, 73.21%CG) of the *S. cattleya* NRRL 8057 genome. As shown in Fig. 1, the TGC, with size of 28.25-Kb and %GC content of 73.61%, is composed of 22 genes in which *thnI* and *thnU* encode for the transcriptional activators of expression of the *thnH*, *J*, *K*, *L*, *M*, *N*, *O*, *P*, and *Q* genes*,* while the expression of the remaining genes (*thnA-G*, *thnR-V*) are *thnIU*-independent^12^. BlastP analysis of the ThnS sequence against the NCBI database reveals the best homologous sequences (coverage ≥ 50% and similarity ≥ 84%) with metallo-β-lactamase (MBL), fold metallo-hydrolase and the L-ascorbate metabolism protein UlaG, all identified from the *Pseudonocardiaceae* family. Its best homologous sequences within more common bacteria, especially Enterobacteriaceae species, are also all annotated as MBL fold metallo-hydrolase with homologies ranging from 27% coverage and 34% identity with *Serratia ficaria* and 79% coverage and 24.15% identity with *Escherichia coli* (Suppl. Table S1).

### Phylogenetic relationship of the ThnS enzyme with bacterial β-lactamases

**Figure 2:**
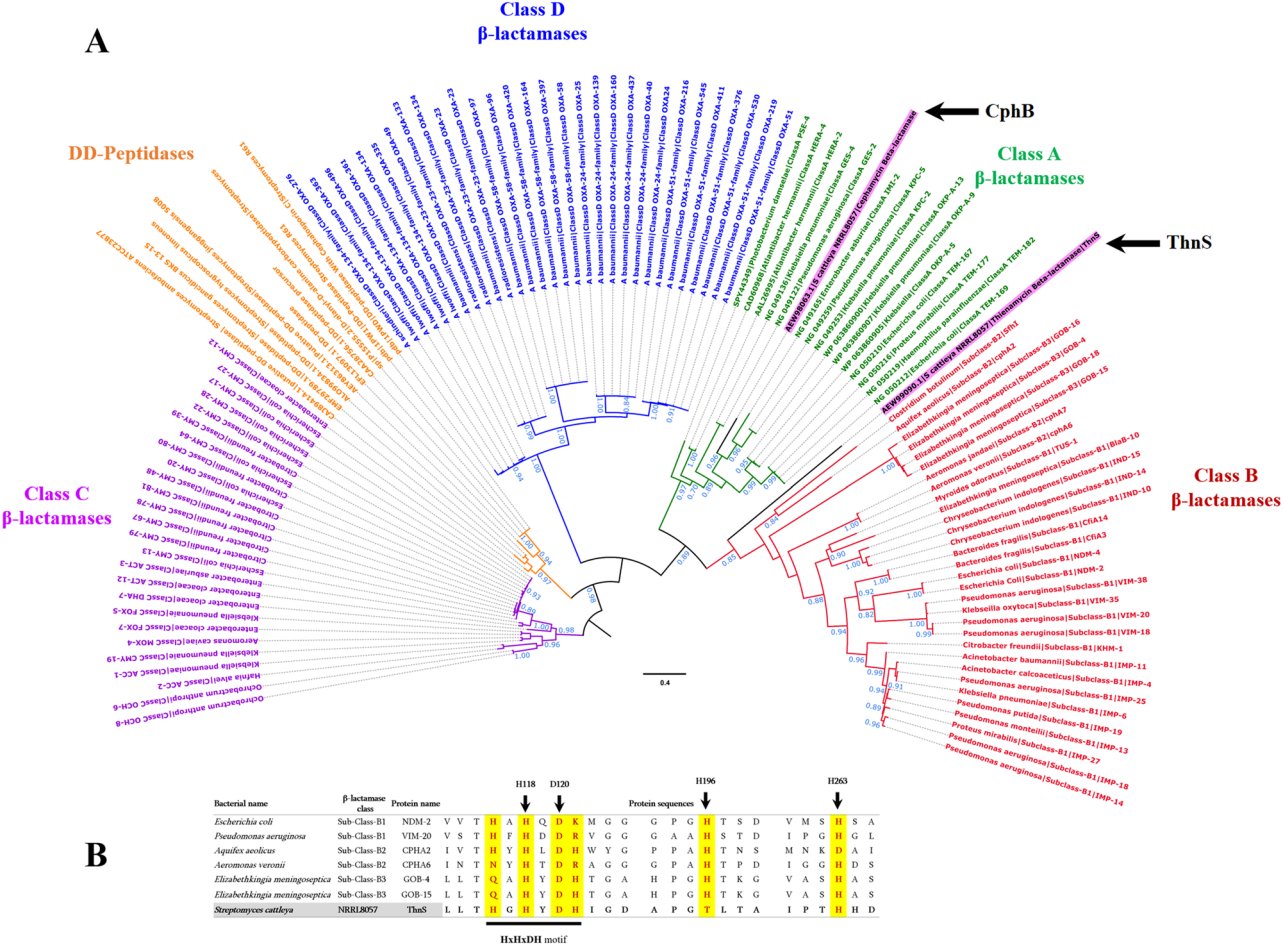
Phylogenetic relationship of ThnS with bacterial β-lactamases. (**A**) phylogenetic tree analysis of the ThnS protein with described bacterial β-lactamase sequences; (**B**) protein alignment of ThnS with class B β-lactamase sequences exhibiting the conserved motif and residues of this class. The phylogenetic tree was inferred using the maximum-likelihood method in FastTree then displayed using the FigTree software v1.4.4 (http://tree.bio.ed.ac.uk/software/figtree/).

The inferred phylogenetic tree of the ThnS sequence with the four bacterial classes of β-lactamases (A, B, C, and D) shows that the ThnS enzyme is closely related to the bacterial class B β-lactamases (Fig. 2A). This membership is confirmed by sequence alignment with the three sub-classes (B1, B2, B3) highlighting the conserved “HxHxDH” motif and H196, H263 residues, that are specific to this metallo-β-lactamase (MBL) superfamily group (Fig. 2B). Moreover, three-dimensional (3D) structural analysis of the ThnS protein model from the Phyre2 investigator database confirms with 100% confidence and more than 81% coverage that ThnS was similar to the crystal structure of an uncharacterised metallo protein from *Vibrio cholerae* with β-lactamase-like fold (Phyre2 ID: c3bv6D; Uniprot ID: Q9KMS2).

### β-lactam-hydrolysing activity

**Figure 3:**
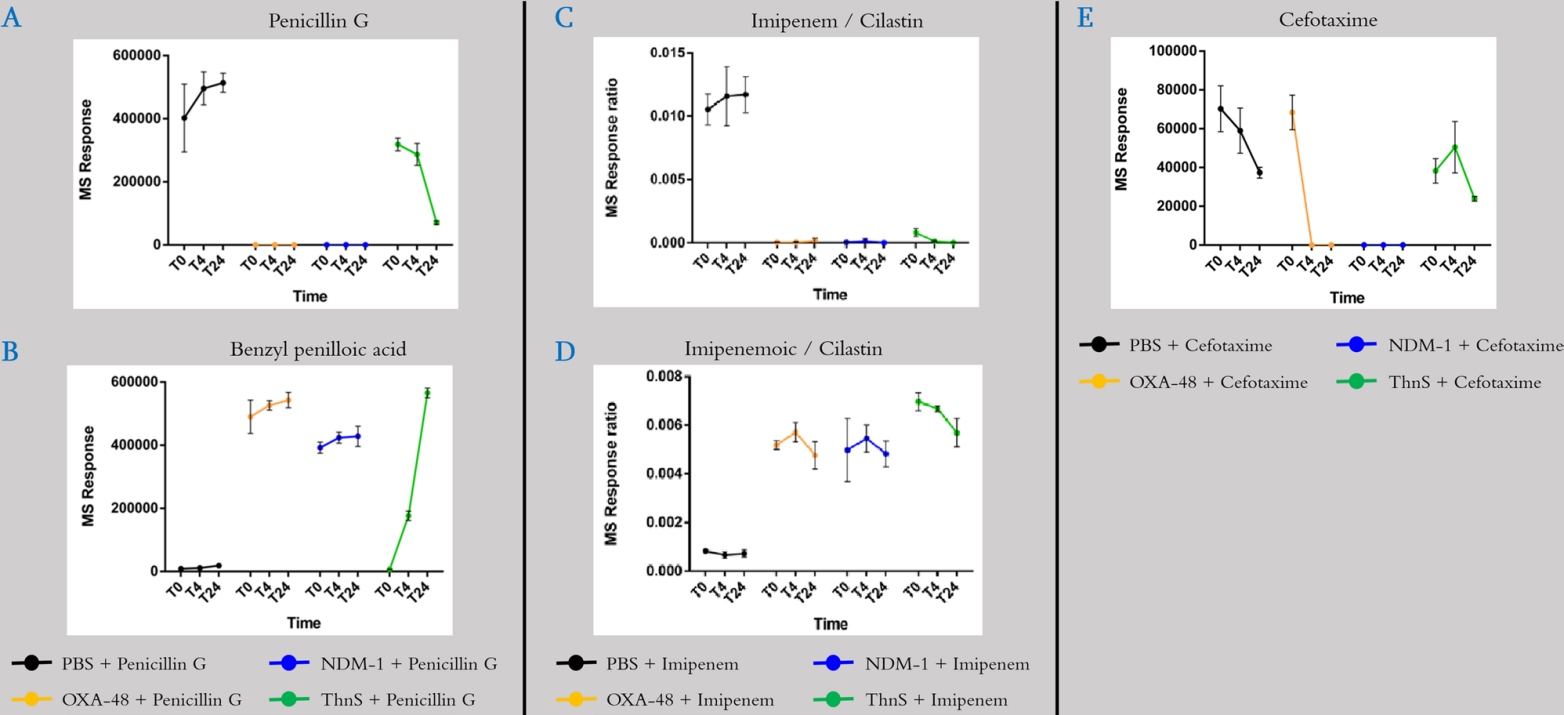
Monitoring the degradation of β-lactam drugs by the expressed ThnS enzyme compared to known bacterial β-lactamases (OXA-48 and NDM-1). (**A**) detection of Penicillin G over 24 h in presence or absence of the tested enzymes; (**B**) detection of the penicillin G metabolite (benzyl penilloic acid) when Penicillin G is incubated with the tested enzymes over 24 h; (**C**) monitoring the amount of imipenem when incubated with the tested enzymes over 24 h; (**D**) monitoring the amount of imipenem metabolite (imipenemoic acid) when imipenem is incubated with the tested enzymes over 24 h; (**E**) monitoring the amount of cefotaxime when incubated with the tested enzymes over 24 h.

To evaluate the enzymatic activity of ThnS, this latter and two bacterial β-lactamases including class D OXA-48 and class B NDM-1 carbapenemases were expressed and tested against different antibiotic substrates including nitrocefin, penicillin G, cefotaxime, and imipenem. Interestingly, ThnS was unable to hydrolyze the nitrocefin substrate while both bacterial β-lactamases (OXA-48 and NDM-1) significantly hydrolyse this substrate (data not shown). However, as presented in Fig. 3A,B, the ThnS enzyme degrades penicillin G, as observed for OXA-48 and NDM-1 enzymes, but more slowly during the 24 h test, suggesting a lower affinity toward penicillin G. Regarding imipenem hydrolysis, the ThnS activity was comparable with both OXA-48 and NDM-1 carbapenemases (Fig. 3C). ThnS here appears to be more active against this drug since the measured amount of imipenem metabolite (imipenemoic acid) after 24 h of incubation was higher for ThnS compared to the two enzyme controls (Fig. 3D). Compared to both bacterial β-lactamases, the hydrolysis of cefotaxime by ThnS was detected only after 24 h and appears then less active against this cephalosporin compared to the enzyme controls, also suggestive of a lower affinity toward cephalosporin antibiotic (Fig. 3E).

### Degradation activity on ascorbic acid

**Figure 4:**
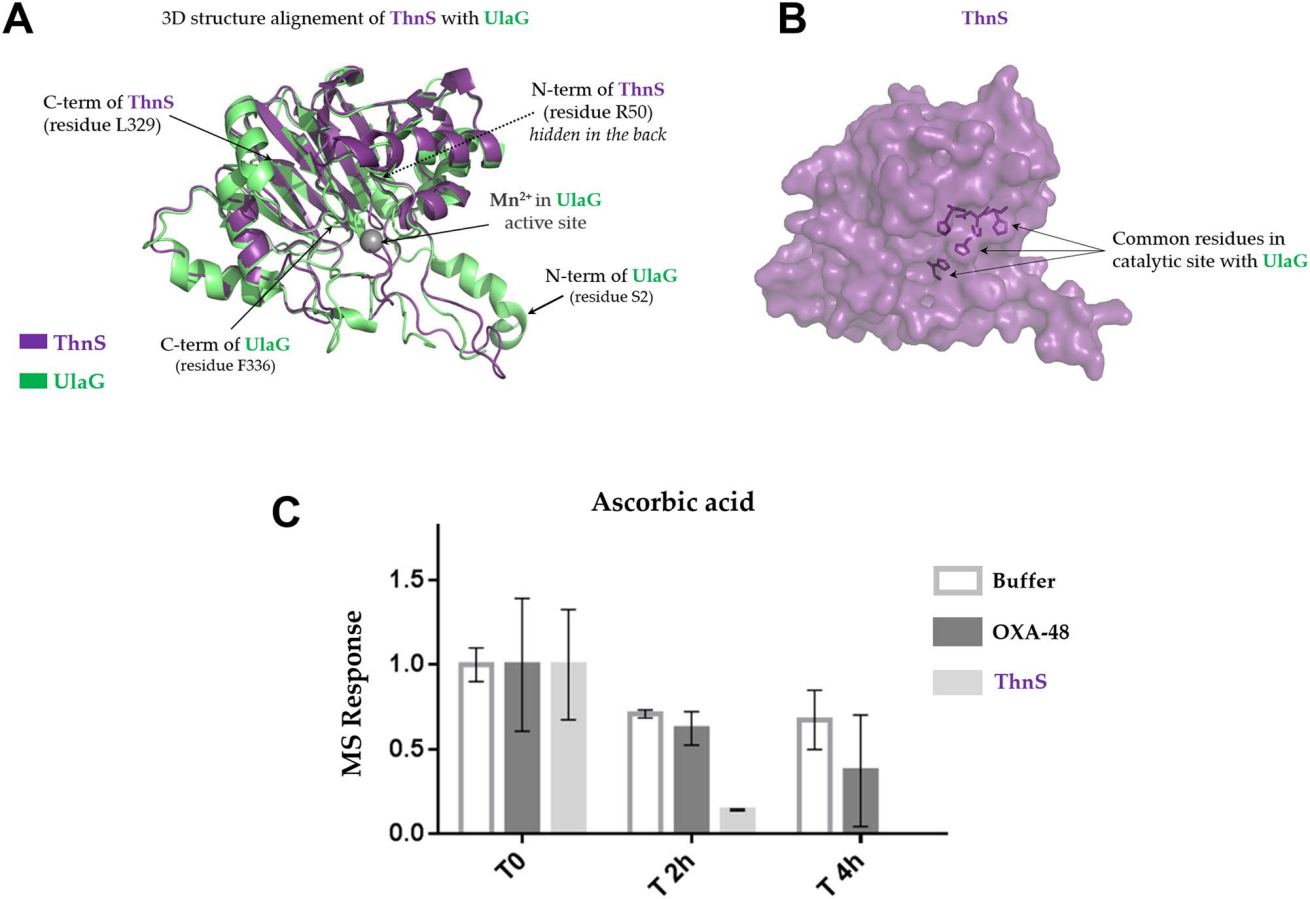
3D model of the ThnS enzyme and monitoring ascorbic acid degradation by ThnS. (**A**) 3D structure comparison of the ThnS Phyre2 model and the UlaG structure (PDB: 2WYM); (**B**) 3D model of ThnS showing common residues in the catalytic site with the UlaG enzyme; structure alignment and visualisation were performed using the PyMOL 1.8.6.0 software (https://github.com/schrodinger/pymol-open-source). (**C**) monitoring ascorbic acid degradation by ThnS using LC–MS. OXA-48 carbapenemase enzyme is used here as a negative enzyme control.

Based on BlastP analyses (presented above), the ThnS enzyme exhibited significant homology with UlaG, a manganese-dependent metallo-β-lactamase enzyme involved in L-ascorbate metabolism through the degradation of ascorbic acid^13^. Interestingly, it has been suggested that the UlaG precursor shared the RNA metabolising function with the last common ancestor enzyme and its descendants have a wide functional activities and phylogenetic distribution^13^. Figure 4A, ThnS enzyme shows 3D structure similarity with UlaG protein from *E. coli* and the same amino acid residues in their catalytic site (Fig. 4B). Thus, based on similarity of protein sequence and 3D structure with UlaG enzymes, ThnS activity against ascorbic acid was tested and monitored by LC–MS. As expected, compared to the control enzyme (OXA-48), ThnS significantly degrades ascorbic acid substrate after two hours of incubation, and after four hours the substrate was no longer detected (Fig. 4C).

### Nuclease and ribonuclease activity

**Figure 5:**
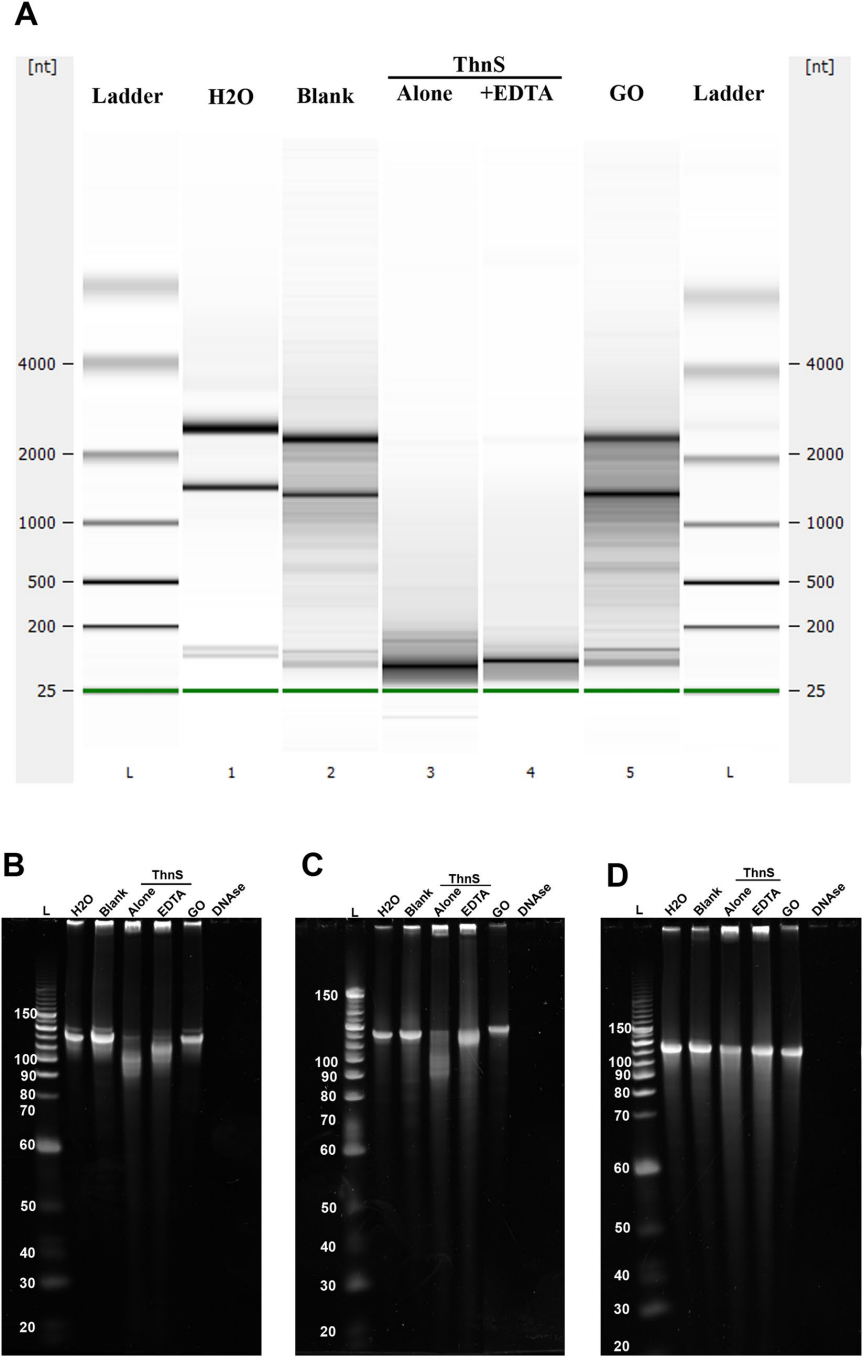
Nuclease and ribonuclease activities of the purified ThnS enzyme. (**A**) Degradation of bacterial total RNAs by the ThnS enzyme in the presence and absence of metallo-β-lactamase inhibitor (EDTA). GO (Glycine Oxidase) is used here as negative control enzyme; (**B**) Nuclease activity of ThnS on dsDNA; (**C**) Nuclease activity of ThnS on forward ssDNA ; (**D**) Nuclease activity of ThnS on reverse ssDNA. The ThnS activity was tested in the presence and absence of a metallo-β-lactamase inhibitor (EDTA). Glycine Oxidase (GO) and DNAse enzymes were used here as negative and positive controls, respectively. “Blank” refers to the empty vector eluted under the same conditions as that of the expressed ThnS enzyme.

Previously reported in the literature, MBL fold proteins can exhibit different promiscuous enzymatic activities such as nuclease and/or ribonuclease activity^14,15^. Here, in addition to the detected β-lactam-hydrolysing and ascorbic acid degradation activities of this enzyme, its nuclease and ribonuclease activities were investigated. Interestingly, after two hours incubating ThnS with extracted *E. coli* RNA, the extracted *E. coli RNA* was specifically and completely hydrolysed in the presence or absence of a metallo-β-lactamase inhibitor (i.e., chelating EDTA agent) while using negative control enzyme (Glycine oxidase) did not (Fig. 5A). Moreover, synthetic single and double-stranded DNA was incubated with ThnS to evaluate its DNAse activity. Interestingly, while the double-stranded DNA was not affected by the ThnS enzyme (Fig. 5B) as observed for the positive control enzyme used (bacterial DNAse), both forward single DNA (ssDNA Fwd)(Fig. 5C) and reversed single DNA (ssDNA Rev)(Fig. 5D) were degraded by ThnS and the tested metallo-β-lactamase inhibitor did not affect its nuclease activity. As expected, the negative control enzyme (GO) showed no DNAse activity.

## Discussion

Natural β-lactam antibiotics such as penicillin, cephalosporin C, or thienamycin^6,16^ are expressed as poisons in the ancestral battle of microorganisms to colonise and master their ecosystems. In this context, β-lactamase enzymes appeared as antidotes to β-lactams produced by bacteria for their self-protection against these bioactive compounds. This suggest that like toxin/antitoxin systems, genes encoding for β-lactams and β-lactamases have always co-evolved within the NRPS/PKS clusters in microorganisms and we can speculate that the human massive use and misuse of antibiotics such as β-lactams during the last century has changed this co-evolution, leading to need for bacteria to acquired β-lactamases. Resistance to carbapenems, especially to imipenem, the N-formimidoyl derivative of thienamycin, now represents one of the biggest concerns in treating infections caused by multidrug resistant bacteria^17^. In this study, we showed that the *thnS* gene, part of the thienamycin gene cluster responsible for the biosynthesis and expression of the thienamycin drug by the *S. cattleya* NRRL8057 isolate^11^, is closely related to bacterial class B MBL enzymes. We demonstrated a β-lactam-hydrolysing activity of the ThnS enzyme and, interestingly, a specific and more efficient activity against imipenem. So, the presence of the *thnS* gene in this thienamycin gene cluster, demonstrates its antidote role toward the produced thienamycin poison. According to this finding, it is predictable to find that bacteria producing antibiotics from the NRPS/PKS clusters, may harbour within these clusters an antidote protein co-expressed with the bioactive poison. In fact, in addition to this poison/antidote system (i.e., thienamycin/ThnS β-lactamase) within the *S. cattleya* NRRL8057 genome, another biosynthesis gene cluster, the cephamycin gene cluster (CGC), responsible for the synthesis of isopenicillin N and cephamycin antibiotics, has been described in this bacterium^10^ and near the CGC, a Class A-like β-lactamase gene (*cphB*), phylogenetically related to extended-spectrum-β-lactamase (ESBL) can be identified (Suppl. fig. S1). In other bacterial pathogens such as *Erwinia carotovora*, a producer of carbapenem antibiotic from a “Car” antibiotic biosynthesis gene cluster in its genome, a same poison/antidote system has been identified in which the antidote proteins correspond to an operon *carFG* encoding for an efflux pump, conferring an intrinsic resistance mechanism^12^. The same CarFG-like efflux pump has also been identified in two *Serratia*, located within the antibiotic biosynthesis gene cluster^12^. In conclusion, the ThnS enzyme appears as a class B metallo-β-lactamase enzyme with an essential antidote role for *S. cattleya* bacterium toward its biosynthesised thienamycin poison.

## Materials and methods

### Phylogeny of the ThnS protein

To investigate the phylogenetic relationship of the ThnS protein from the *Streptomyces cattleya* NRRL8057 genome with known bacterial β-lactamases, representative protein sequences of the four described β-lactamase classes (i.e. A, B, C, and D) were retrieved from the ARG-ANNOT database^18^ and pooled together to infer a phylogenetic tree. Moreover, some DD-peptidase sequences, more related to class C β-lactamase were retrieved from the NCBI database and included in the phylogenetic tree analysis. All protein sequences were aligned using Mafft^19^; gaps within alignment were removed using Trimal^20^ and the phylogenetic tree was inferred using the maximum-likelihood method in FastTree^21^, then displayed using the FigTree software v1.4.4 (http://tree.bio.ed.ac.uk/software/figtree/).

### Recombinant protein expression and purification

The whole tested protein including peptide signals of ThnS, NDM-1 (Ac. n° HQ328085), and OXA-48 (Ac. n° QSS32949.1) were designed to include a Strep-tag at the N-terminus and the corresponding nucleic acid sequence were optimised for expression in *Escherichia coli*. Its corresponding genes were synthetised by GenScript (Piscataway, NJ, USA) and ligated between the NdeI and NotI restriction sites of a pET24a( +) plasmid. *E. coli* BL21(DE3)-pGro7/GroEL (Takara, Kyoto, Japan), grown in ZYP-5052 media were used for the expression of the recombinant protein. When the culture reached an OD_600 nm_ = 0.6 at 37 °C, the temperature was decreased to 20 °C and L-arabinose (0.2% m/v) was added in order to induce the expression of chaperones. As we previously reported, after 20 h, cells were harvested by centrifugation (5,000 g, 30 min, 4 °C) and the pellet was resuspended in washing buffer (50 mM Tris pH 8, 300 mM NaCl) then stored at -80 °C overnight^22^. Frozen *E. coli* were thawed and incubated on ice for one hour after the addition of lysozyme, DNAse I and PMSF (phenylmethylsulfonyl fluoride) to final concentrations of 0.25 mg/mL, 10 µg/mL and 0.1 mM, respectively^22^. Partially lysed cells were then disrupted by three consecutive cycles of sonication (30 s, amplitude 45) performed on a Q700 sonicator system (QSonica). Cellular debris were discarded following centrifugation (10,000 g, 20 min, 4 °C)^22^. The ThnS protein was purified with an ÄKTA avant system (GE Healthcare, Chicago, IL, USA) using Strep-tag affinity chromatography (wash buffer: 50 mM Tris pH 8, 300 mM NaCl, and Elution buffer: 50 mM Tris pH 8, 300 mM NaCl, 2.5 mM desthiobiotin) on a 5 mL StrepTrap HP column (GE Healthcare). Fractions containing the protein of interest were pooled. Protein purity was assessed using 12.5% SDS-PAGE analysis (Coomassie staining)^22^. Protein expression was confirmed by performing MALDI-TOF MS analysis on gel bands previously obtained by SDS-PAGE. Protein concentrations were measured using a Nanodrop 2000c spectrophotometer (Thermo Scientific, Madison, WI, USA)^22^.

### Monitoring imipenem, penicillin G, cefotaxime and ascorbic acid degradation using liquid chromatography-mass spectrometry (LC–MS)

A stock solution of 10 mg/ml of Imipenem and Cilastatin was freshly prepared in water from the perfusion mixture of both compounds (500 mg/500 mg; Panpharma, Luitre, France). Stock solutions of Penicillin G, Cefotaxime and ascorbic acid were prepared at 1 mg/mL in water from pure standards (Sigma Aldrich, Lyon, France). 100 μL of ThnS enzyme aliquots at 1 mg/mL were spiked with Imipenem/Cilastatin or Penicillin G or Cefotaxime or ascorbic acid at final concentrations of 10 μg/ml, then incubated at room temperature. Negative controls consisted of PBS 1 × spiked with the antibiotics or ascorbic acid alone. Triplicate samples were prepared and for each replicate, 30 μL of solution was collected at 0, 4 and 24 h. 70 μL of acetonitrile was then added to each sample, and tubes were vortexed for 10 min at 16,000 g in order to precipitate proteins. The clear supernatant was collected for analysis using an Acquity I-Class UPLC chromatography system connected to a Vion IMS Qtof ion mobility-quadrupole-time of flight mass spectrometer as described previously^22,23^. To summarise, samples were injected into a reverse phase column (Acquity BEH C18 1.7 μm 2.1 × 50 mm, Waters), and compounds were ionised in the positive mode using a Zspray electrospray ion source. Ions were monitored using a high-definition MS (E) data independent acquisition method. 4D peaks, corresponding to chromatographic retention time, ion mobility drift time and parents/fragments masses, were then collected from raw data using UNIFI software (version 1.9.3, Waters). The lactam rings of Imipenem and Penicillin G can be hydrolysed respectively to Imipenemoic acid and benzyl penilloic acid; these structures were targeted by m/z ratio, retention time and drift time. The hydrolysed forms of Cefotaxime and ascorbic acid were not monitored.

### Structural prediction and alignment of ThnS

The protein sequence of ThnS was submitted to the Phyre2 web portal (http://www.sbg.bio.ic.ac.uk/phyre2/html/page.cgi?id=index) for protein modelling, prediction and analysis. The resulting pdb model was visualised and structurally aligned with the published structure of UlaG from *Escherichia coli* (PDB : 2WYM)^24^ using the PyMOL 1.8.6.0 software (https://github.com/schrodinger/pymol-open-source).

### Nuclease and ribonuclease activity testing

Enzymatic reactions were performed by incubating each polynucleotide or polyribonucleotide (1 µg) with 10 µg of purified ThnS enzyme alone or in the presence of a metallo-β-lactamase inhibitor (10 mM of EthyleneDiamineTetraacetic Acid, EDTA) and using a final volume of 15 µl at 37 °C for two hours. Single-stranded DNAs were synthetic polynucleotides (Suppl. Table S2); double-stranded DNA (130-bp) was obtained by annealing positive and negative single-stranded DNAs (130-bp) in a thermocycler at temperatures decreasing from 95 °C to 25 °C over the course of two hours. The enzymatic reactions were conducted in CutSmart Buffer (New England Biolabs, 50 mM Potassium Acetate, 20 mM Tris–acetate, 10 mM Magnesium Acetate, 100 µg/ml BSA pH 7.9) and using a final volume of 15 µl at 37 °C for two hours. After incubation, the material was loaded onto denaturing PolyAcrylamide Gel Electrophoresis (dPAGE) at 12%. A negative enzyme control, *Bacillus subtilis* Glycine Oxidase (GO), was expressed and purified under the same conditions as ThnS and tested. A Turbo DNAse enzyme was also used as positive control enzyme at concentration of 1 U. To evaluate ribonuclease activity, the *Escherichia coli* RNA was purified using the RNeasy columns (Invitrogen, USA). The integrity of the RNA preparations was tested on a Bioanalyzer 2100 (Agilent), using Agilent RNA 6000 Pico LabChip and shown a high integrity with RNA Integrity Number (RIN) = 10. The enzymatic reactions were performed by incubating RNA (1 µg) with 10 µg of purified ThnS enzyme alone and in the presence of a metallo-β-lactamase inhibitor, in CutSmart Buffer and using a final volume of 15 µl at 37 °C for two hours. After incubation, RNA-hydrolysing activity was visualised on the RNA 6000 Pico LabChip (Agilent 2100 Bioanalyzer). The *B. subtilis* Glycine oxidase was also used as negative control enzyme.

## Supplementary Information

Supplementary Information.
