# Supplementary material for: A metallo-β-lactamase enzyme for internal detoxification of the antibiotic thienamycin
Source: Sci Rep. 2021 May 12;11:10062. doi: 10.1038/s41598-021-89600-x (PMC8115136; doi:10.1038/s41598-021-89600-x)
Supplement: Supplementary file 1 — Supplementary Information. [file 41598_2021_89600_MOESM1_ESM.doc]

**A metallo-β-lactamase enzyme for internal detoxification of the antibiotic thienamycin**

Seydina M. Diene^1,3^, Lucile Pinault^2,3^, Sophie Alexandra Baron^2,3^, Saïd Azza^1,3^, Nicholas Armstrong^2,3^, Linda Hadjadj^1,3^, Eric Chabrière^1,3^, Jean-Marc Rolain^1,3^, Pierre Pontarotti^1,3,4^, Didier Raoult^1,3^*****

*** Corresponding author**: Prof. Didier Raoult

**Address**: MEPHI, IHU-Méditerranée Infection, 19-21 Boulevard Jean Moulin, 13005 Marseille

**E-mail:** [didier.raoult@gmail.com](mailto:didier.raoult@gmail.com)

**Phone**: (+33) 4 13 73 24 01.

**Fax:** (+33) 4 13 73 24 02


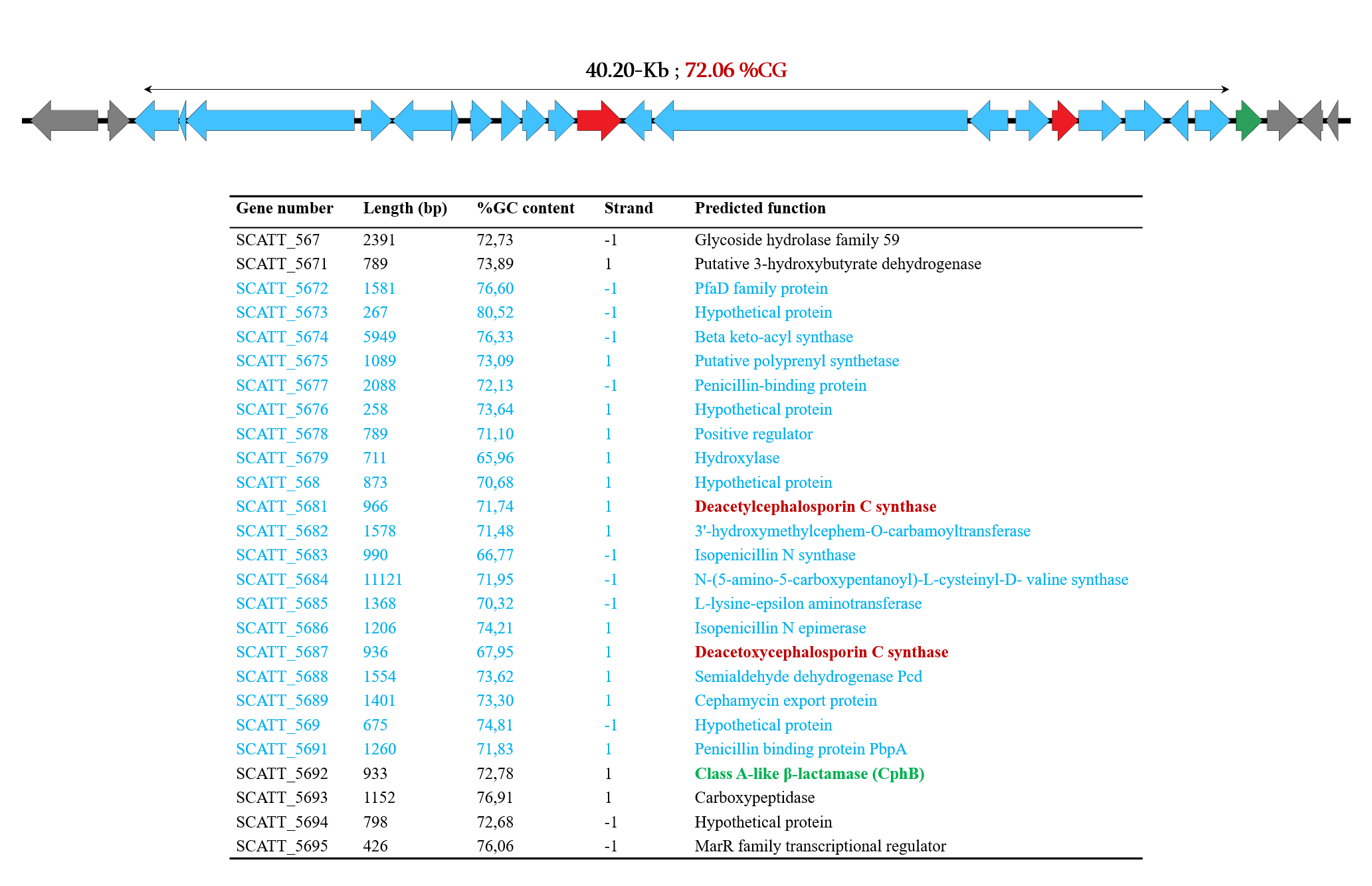


**Suppl. Figure S1:** Cephamycin gene cluster from the linear chromosome of *Streptomyces cattleya* NRRL8057.

**Suppl. Table S1**: The 100 best hits of ThnS protein of *S. cattleya* within Enterobacteriaceae species

| **Bacterial names** | **Predicted functions** | **Query**  **Cover** | **% aa**  **identity** | **E-value** | **Max Score** | **Total Score** | **Accession number** |
| --- | --- | --- | --- | --- | --- | --- | --- |
| *Serratia ficaria* | MBL fold metallo-hydrolase | 27% | 34,02 | 0.029 | 44.7 | 44.7 | WP_061794593.1 |
| *Serratia sp.* 1D1416 | MBL fold metallo-hydrolase | 27% | 34,02 | 0.027 | 44.7 | 44.7 | WP_129544420.1 |
| *Candidatus Regiella insecticola* | hydroxyacylglutathione hydrolase | 37% | 33,06 | 0.080 | 43.1 | 43.1 | WP_006707722.1 |
| *Cronobacter universalis* NCTC9529 | Outer membrane protein romA | 42% | 32,62 | 1,00E-06 | 58.2 | 58.2 | CCK16934.1 |
| *Gammaproteobacteria* | MBL fold metallo-hydrolase | 42% | 32,62 | 9,00E-07 | 58.5 | 58.5 | WP_032805126.1 |
| *Enterobacter hormaechei* | MBL fold metallo-hydrolase | 41% | 31,62 | 3,00E-06 | 56.6 | 56.6 | WP_047727073.1 |
| *Pantoea sp.* ICBG985 | MBL fold metallo-hydrolase | 41% | 31,62 | 4,00E-06 | 56.6 | 56.6 | WP_104093105.1 |
| *Escherichia coli* | metal-dependent hydrolase | 34% | 31,58 | 0.002 | 45.8 | 45.8 | WP_152924043.1 |
| *Enterobacterales* | MBL fold metallo-hydrolase | 56% | 31,25 | 3,00E-05 | 53.9 | 53.9 | WP_047955838.1 |
| *Enterobacter hormaechei* | MBL fold metallo-hydrolase | 56% | 30,77 | 3,00E-05 | 53.9 | 53.9 | WP_047063011.1 |
| *Klebsiella pneumoniae* | MBL fold metallo-hydrolase | 42% | 30,5 | 2,00E-04 | 51.2 | 51.2 | WP_142483169.1 |
| *Klebsiella pneumoniae* | metal-dependent hydrolase | 38% | 28,91 | 0.049 | 41.6 | 41.6 | WP_129498616.1 |
| *Pantoea sp.* Sc1 | MBL fold metallo-hydrolase | 53% | 27,57 | 0.029 | 44.7 | 44.7 | WP_009089812.1 |
| *Pantoea vagans* | MBL fold metallo-hydrolase | 53% | 27,57 | 0.031 | 44.7 | 44.7 | WP_140028630.1 |
| *Raoultella terrigena* | hypothetical protein | 34% | 27,5 | 3,00E-04 | 50.8 | 50.8 | WP_148250501.1 |
| *Xenorhabdus ehlersii* | hydroxyacylglutathione hydrolase | 50% | 27,38 | 0.003 | 47.4 | 47.4 | WP_099133803.1 |
| *Citrobacter freundii complex* | MBL fold metallo-hydrolase | 46% | 26,95 | 0.027 | 45.1 | 45.1 | WP_103856653.1 |
| *Serratia marcescens* | MBL fold metallo-hydrolase | 40% | 26,81 | 0.083 | 42.4 | 42.4 | WP_154643002.1 |
| *Xenorhabdus* | hydroxyacylglutathione hydrolase | 55% | 26,78 | 0.10 | 42.7 | 42.7 | WP_047768201.1 |
| *Serratia fonticola* | MBL fold metallo-hydrolase | 55% | 26,56 | 0.002 | 48.1 | 48.1 | WP_161712442.1 |
| *Serratia fonticola* | MBL fold metallo-hydrolase | 55% | 26,56 | 0.002 | 48.1 | 48.1 | WP_098931920.1 |
| *Serratia fonticola* | MBL fold metallo-hydrolase | 55% | 26,56 | 0.002 | 48.1 | 48.1 | WP_161739112.1 |
| *Serratia fonticola* | MBL fold metallo-hydrolase | 55% | 26,56 | 0.004 | 47.4 | 47.4 | WP_024531222.1 |
| *Serratia fonticola* | MBL fold metallo-hydrolase | 55% | 26,56 | 0.005 | 47.0 | 47.0 | WP_024486482.1 |
| *Serratia sp.* 14-2641 | MBL fold metallo-hydrolase | 55% | 26,56 | 0.004 | 47.4 | 47.4 | WP_065684269.1 |
| *Serratia sp.* 3ACOL1 | MBL fold metallo-hydrolase | 55% | 26,56 | 0.007 | 46.6 | 46.6 | WP_121607124.1 |
| *Klebsiella variicola* | MBL fold metallo-hydrolase | 37% | 26,52 | 1,00E-04 | 52.4 | 52.4 | WP_052749769.1 |
| *Klebsiella variicola* | hypothetical protein | 37% | 26,52 | 2,00E-04 | 51.2 | 51.2 | WP_177332806.1 |
| *Klebsiella variicola* | MBL fold metallo-hydrolase | 37% | 26,52 | 2,00E-04 | 51.2 | 51.2 | WP_049158013.1 |
| *Pantoea sp.* | MBL fold metallo-hydrolase | 53% | 26,52 | 0.050 | 43.9 | 43.9 | WP_167430756.1 |
| *Pantoea agglomerans* | MBL fold metallo-hydrolase | 53% | 26,52 | 0.044 | 44.3 | 44.3 | WP_154928242.1 |
| *Pantoea anthophila* | MBL fold metallo-hydrolase | 53% | 26,52 | 0.017 | 45.4 | 45.4 | WP_046101955.1 |
| *Pantoea anthophila* | MBL fold metallo-hydrolase | 53% | 26,52 | 0.047 | 43.9 | 43.9 | WP_140922917.1 |
| *Pantoea sp.* ARC270 | MBL fold metallo-hydrolase | 53% | 26,52 | 0.047 | 43.9 | 43.9 | WP_111207564.1 |
| *Plautia stali* | MBL fold metallo-hydrolase | 53% | 26,52 | 0.092 | 43.1 | 43.1 | WP_058957304.1 |
| *unclassified Pantoea* | MBL fold metallo-hydrolase | 53% | 26,52 | 0.042 | 44.3 | 44.3 | WP_150029506.1 |
| *Pantoea sp.* | MBL fold metallo-hydrolase | 59% | 26,37 | 8,00E-04 | 49.7 | 49.7 | WP_089561420.1 |
| *Pantoea brenneri* | MBL fold metallo-hydrolase | 59% | 26,37 | 5,00E-04 | 50.1 | 50.1 | WP_069728556.1 |
| *Pantoea sp.* 111 | MBL fold metallo-hydrolase | 59% | 26,37 | 5,00E-04 | 50.1 | 50.1 | WP_159223463.1 |
| *Pantoea sp.* 3.5.1 | MBL fold metallo-hydrolase | 59% | 26,37 | 6,00E-04 | 49.7 | 49.7 | WP_031374638.1 |
| *Serratia sp.* | MBL fold metallo-hydrolase | 40% | 26,09 | 0.055 | 43.9 | 43.9 | WP_038877061.1 |
| *Serratia marcescens* | MBL fold metallo-hydrolase | 40% | 26,09 | 0.056 | 43.9 | 43.9 | WP_047730519.1 |
| *Serratia marcescens* E28 | MBL fold metallo-hydrolase | 40% | 26,09 | 0.056 | 43.9 | 43.9 | WP_061872623.1 |
| *Serratia marcescens* | MBL fold metallo-hydrolase | 40% | 26,09 | 0.058 | 43.9 | 43.9 | WP_080491516.1 |
| *Serratia marcescens* | MBL fold metallo-hydrolase | 40% | 26,09 | 0.058 | 43.9 | 43.9 | WP_110663981.1 |
| *Serratia marcescens* | MBL fold metallo-hydrolase | 40% | 26,09 | 0.059 | 43.9 | 43.9 | WP_110610967.1 |
| *Serratia marcescens* | MBL fold metallo-hydrolase | 40% | 26,09 | 0.067 | 43.5 | 43.5 | WP_110684685.1 |
| *Pantoea conspicua* | MBL fold metallo-hydrolase | 61% | 26,07 | 0.013 | 45.8 | 45.8 | WP_094119959.1 |
| *Enterobacteriales bacterium* SAP-6 | MBL fold metallo-hydrolase | 56% | 26,04 | 0.094 | 43.1 | 43.1 | WP_162365231.1 |
| *Escherichia coli* | metal-dependent hydrolase | 51% | 26,04 | 0.001 | 47.4 | 47.4 | WP_151040253.1 |
| *Serratia fonticola* | MBL fold metallo-hydrolase | 55% | 26,04 | 0.011 | 45.8 | 45.8 | WP_021806779.1 |
| *Serratia fonticola* | MBL fold metallo-hydrolase | 55% | 26,04 | 0.012 | 45.8 | 45.8 | WP_021181030.1 |
| *Serratia fonticola* | MBL fold metallo-hydrolase | 55% | 26,04 | 0.012 | 45.8 | 45.8 | WP_179246581.1 |
| *Serratia fonticola* | L-ascorbate metabolism protein UlaG (β-lactamase superfamily) | 55% | 26,04 | 0.013 | 45.8 | 45.8 | RDL27817.1 |
| *Xenorhabdus thuongxuanensis* | hydroxyacylglutathione hydrolase | 57% | 26,04 | 0.008 | 46.2 | 46.2 | WP_074019358.1 |
| *Pantoea deleyi* | MBL fold metallo-hydrolase | 53% | 25,56 | 0.019 | 45.4 | 45.4 | WP_140917264.1 |
| *Serratia fonticola* | MBL fold metallo-hydrolase | 55% | 25,52 | 0.039 | 44.3 | 44.3 | WP_141132439.1 |
| *Serratia fonticola* | metal-dependent hydrolase | 55% | 25,52 | 0.041 | 44.3 | 44.3 | VEI71929.1 |
| *Serratia fonticola* | hypothetical protein HAP32_00321 | 55% | 25,52 | 0.094 | 43.1 | 43.1 | QIP89804.1 |
| *Serratia fonticola* | MBL fold metallo-hydrolase | 55% | 25,52 | 0.096 | 43.1 | 43.1 | WP_166732771.1 |
| *unclassified Serratia* | MBL fold metallo-hydrolase | 55% | 25,52 | 0.009 | 46.2 | 46.2 | WP_025121886.1 |
| *Xenorhabdus eapokensis* | hydroxyacylglutathione hydrolase | 57% | 25,52 | 0.001 | 48.5 | 48.5 | WP_074024680.1 |
| *Serratia oryzae* | MBL fold metallo-hydrolase | 61% | 25,47 | 0.011 | 45.8 | 45.8 | WP_076941307.1 |
| *Xenorhabdus beddingii* | hydroxyacylglutathione hydrolase | 58% | 25,47 | 0.025 | 44.7 | 44.7 | WP_086112368.1 |
| *Pantoea agglomerans* | MBL fold metallo-hydrolase | 53% | 25,41 | 0.069 | 43.5 | 43.5 | WP_010258798.1 |
| *Escherichia coli* | metal-dependent hydrolase | 39% | 25,38 | 0.002 | 45.4 | 45.4 | WP_152934389.1 |
| *Serratia sp.* | MBL fold metallo-hydrolase | 53% | 25,27 | 0.059 | 43.9 | 43.9 | WP_033634894.1 |
| *Serratia sp.* | MBL fold metallo-hydrolase | 53% | 25,27 | 0.064 | 43.5 | 43.5 | WP_033647688.1 |
| *Serratia marcescens* | MBL fold metallo-hydrolase | 53% | 25,27 | 0.016 | 45.4 | 45.4 | WP_103773577.1 |
| *Serratia marcescens* | MBL fold metallo-hydrolase | 53% | 25,27 | 0.017 | 45.4 | 45.4 | WP_079656564.1 |
| *Serratia marcescens* | MBL fold metallo-hydrolase | 53% | 25,27 | 0.018 | 45.4 | 45.4 | WP_060421409.1 |
| *Serratia marcescens* | MBL fold metallo-hydrolase | 53% | 25,27 | 0.030 | 44.7 | 44.7 | WP_094859901.1 |
| *Serratia marcescens* | MBL fold metallo-hydrolase | 53% | 25,27 | 0.040 | 44.3 | 44.3 | WP_060452582.1 |
| *Serratia marcescens* | MBL fold metallo-hydrolase | 53% | 25,27 | 0.055 | 43.9 | 43.9 | WP_164104824.1 |
| *Serratia marcescens* | MBL fold metallo-hydrolase | 53% | 25,27 | 0.056 | 43.9 | 43.9 | WP_049194715.1 |
| *Serratia marcescens* | MBL fold metallo-hydrolase | 53% | 25,27 | 0.056 | 43.9 | 43.9 | WP_101427028.1 |
| *Serratia marcescens* | MBL fold metallo-hydrolase | 53% | 25,27 | 0.056 | 43.9 | 43.9 | WP_164134362.1 |
| *Serratia marcescens* | MBL fold metallo-hydrolase | 53% | 25,27 | 0.057 | 43.9 | 43.9 | WP_033654428.1 |
| *Serratia marcescens* | MBL fold metallo-hydrolase | 53% | 25,27 | 0.058 | 43.9 | 43.9 | WP_103829594.1 |
| *Serratia marcescens* | MBL fold metallo-hydrolase | 53% | 25,27 | 0.059 | 43.9 | 43.9 | WP_158692495.1 |
| *Serratia marcescens* | MBL fold metallo-hydrolase | 53% | 25,27 | 0.060 | 43.9 | 43.9 | WP_086579990.1 |
| *Serratia marcescens* | MBL fold metallo-hydrolase | 53% | 25,27 | 0.063 | 43.9 | 43.9 | WP_128884774.1 |
| *Serratia marcescens* | MBL fold metallo-hydrolase | 53% | 25,27 | 0.063 | 43.5 | 43.5 | WP_161764509.1 |
| *Serratia marcescens* | MBL fold metallo-hydrolase | 53% | 25,27 | 0.064 | 43.5 | 43.5 | WP_060451629.1 |
| *Serratia marcescens* | MBL fold metallo-hydrolase | 53% | 25,27 | 0.090 | 43.1 | 43.1 | WP_060427413.1 |
| *Serratia marcescens* SM39 | putative phospholipase | 53% | 25,27 | 0.055 | 43.9 | 43.9 | BAO34314.1 |
| *Serratia marcescens* | MBL fold metallo-hydrolase | 51% | 25,14 | 0.088 | 43.1 | 43.1 | WP_049202105.1 |
| *Serratia sp.* BW106 | MBL fold metallo-hydrolase | 55% | 24,74 | 0.10 | 43.1 | 43.1 | WP_099064403.1 |
| *Pantoea vagans* | MBL fold metallo-hydrolase | 53% | 24,44 | 0.065 | 43.5 | 43.5 | WP_095707093.1 |
| *Serratia marcescens* | MBL fold metallo-hydrolase | 59% | 24,38 | 0.058 | 43.9 | 43.9 | WP_060439761.1 |
| *Escherichia coli* | metal-dependent hydrolase | 79% | 24,15 | 5,00E-04 | 49.3 | 49.3 | MQL15180.1 |
| *Salmonella enterica* | L-ascorbate 6-phosphate lactonase | 75% | 24,1 | 0.052 | 43.9 | 43.9 | HAK3502249.1 |
| *Escherichia coli* | MBL fold metallo-hydrolase | 39% | 23,91 | 0.060 | 42.0 | 42.0 | WP_141031851.1 |
| *Pantoea* | MBL fold metallo-hydrolase | 53% | 23,89 | 0.10 | 43.1 | 43.1 | WP_033783293.1 |
| *Pantoea sp.* JKS000250 | MBL fold metallo-hydrolase | 53% | 23,89 | 0.10 | 43.1 | 43.1 | WP_110330392.1 |
| *Pantoea vagans* | MBL fold metallo-hydrolase | 53% | 23,89 | 0.11 | 43.1 | 43.1 | WP_013358105.1 |
| *Citrobacter amalonaticus* | MBL fold metallo-hydrolase | 54% | 23,79 | 0.014 | 45.8 | 45.8 | WP_103776953.1 |
| *Pantoea vagans* | MBL fold metallo-hydrolase | 55% | 23,66 | 0.085 | 43.1 | 43.1 | WP_033732882.1 |
| *Pantoea vagans* | MBL fold metallo-hydrolase | 68% | 23,38 | 0.019 | 45.4 | 45.4 | WP_135908502.1 |
| *Salmonella enterica* | MBL fold metallo-hydrolase | 49% | 21,18 | 0.065 | 43.1 | 43.1 | EAO7613848.1 |

**Suppl. Table S2**: Sequence of DNA synthetic (130 nucleotides) used in enzyme treatments as substrates as a single strand or double strand obtained by annealing forward with reverse synthetic DNA.

| Gene origin | Name | Sequence |
| --- | --- | --- |
| Zamilon  virophage ORF4 | DNA_Synt_Fwd | ATAGAACAACCAAAAAAATATCAAAATCTAGAGATGAATCAAGTGAATCAGAAGAATCTGATAATGAATCTGATAATGAATCCGATGAGGAAGTTGAATCAGAAACTGAGATAGAACCAGTCAAATCTAA |
|  | DNA_Synt_Rev | TTAGATTTGACTGGTTCTATCTCAGTTTCTGATTCAACTTCCTCATCGGATTCATTATCAGATTCATTATCAGATTCTTCTGATTCACTTGATTCATCTCTAGATTTTGATATTTTTTTGGTTGTTCTAT |
